# Feed Components and Egg Allergenicity: Impact of Lupin and Soybean Meal Inclusion on Hen Egg Immunoreactivity (ELISA-Based Study)

**DOI:** 10.3390/foods15071231

**Published:** 2026-04-04

**Authors:** Aneta Tomczak, Piotr Klimowicz, Dorota Piasecka-Kwiatkowska, Łukasz Tomczyk, Magdalena Zielińska-Dawidziak

**Affiliations:** 1 Department of Food Biochemistry and Analysis, Faculty of Food Science and Nutrition, Poznan University of Life Sciences, ul. Wojska Polskiego 28, 62-623 Poznan, Poland; aneta.tomczak@up.poznan.pl (A.T.); piotr.klimowicz@up.poznan.pl (P.K.); dorota.piasecka-kwiatkowska@up.poznan.pl (D.P.-K.); 2 Department of Food Quality and Safety Mangement, Faculty of Food Science and Nutrition, Poznan University of Life Sciences, ul. Wojska Polskiego 28, 62-623 Poznan, Poland; lukasz.tomczyk@up.poznan.pl

**Keywords:** ELISA, food allergy, lupin, soy, egg

## Abstract

Background: Egg proteins are among the most common triggers of allergic reactions. This study aimed to evaluate whether changes in the protein source in hen diets may influence the immunoreactivity of eggs and recognize the ELISA limits in egg allergen detection. Methods: This study used eggs from laying hens fed various feeds (including soy and lupin). Determinations of selected allergens were performed using the immunoenzymatic ELISA method. Results: Studies have shown that including legumes in hens’ diets reduces egg immunoreactivity. The highest detected reactivity using a commercial test for the immunoreactivity of egg albumen was twice as high in whites from hens fed the control diet, and this result was most likely due to the Gal d 1 and Gal d 3 detection. Still, the use of this diet reduced the lysozyme (Gal d 4) content in the egg white by approximately 10%. The applied method did not allow for Gal d 5 determination in the tested eggs. The results of the immunoreactivity of the studied eggs with anti-soy and anti-lupine antibodies were outside the limits of detection. Conclusions: The obtained results confirm that the studied allergen content in eggs can be modified by nutritional factors.

## 1. Introduction

Data from FAOSTAT and the Helgi Library (FAO) show that egg consumption per person in Poland in 2023 was 5.17 kg [1]. The highest average amount eaten in Europe was recorded in the Netherlands (32.45 kg/year/person) [2]. Despite extensive documentation in the literature of eggs’ broad health-promoting properties, they can also be allergenic. A list of egg allergens and their characteristics in presented in Table S1. The Food Safety Commission of Japan (FSCJ) document states that a reference concentration RfD (reference dose) of ~10 µg/g of egg protein in a finished product is used as the labeling threshold [3]. According to the Institute of Food Science and Technology’s VITAL^®^ 3.0 (allergen risk management system), the amount of egg protein that causes a reaction in 1% of the allergy population is ED01 = 0.2 mg of egg protein [4]; while the FAO/WHO states that a reaction will occur in 5% of the allergic population with ED05 = ~2.0 mg of egg protein [5]. EU legislation (e.g., Regulation (EU) No. 1169/2011) on allergen information does not establish a minimum threshold (RfDs) below which the allergen need not be declared. If an egg is used as a food ingredient, it must be declared regardless of the quantity [6]. The information, denoted as ‘may contain’ or ‘possible traces’, refers to the unavoidable risk of unintended allergen cross-contamination, not to situations where egg is an intended food ingredient.

Eggs are included (together with milk) in the most complicated food matrices (or rather raw materials of animal origin) to analyze. This is due to the complexity of the matrix, in which even a small amount of processing influences the analysis results. Denaturation, protein extraction methods, and protein modifications such as glycosylation can significantly and seriously falsify ELISA test results [7]. Thus, analyses of egg allergens are often aimed at detection, not quantification. Due to health consequences, reliable and highly sensitive methods are needed to detect allergenic proteins in eggs. Although ELISA remains the gold standard and first-choice method for quantitative allergen detection, new methods, such as LC-MS/MS, have been developed. These methods are extremely expensive to perform, and yet they are standardized using ELISA [8]. Currently, a wide range of commercial tests based on ELISA methods is available on the market, making the choice difficult. Studies show that different tests can yield different results [9].

There is a large discussion in the scientific literature about the impact of feed changes on egg composition, but few studies have focused on egg allergens (ovalbumin, ovomucoid, lysozyme, ovotransferrin, and α-livetin). Soymeal and lupine, used in hens’ feeding, are also known food allergens and may provoke cross-reactions in people allergic to peanut [10]. Therefore, the study of the allergenicity of eggs after the application of such sources of protein in poultry nutrition is extremely important, interdisciplinary, and modern. Research from recent years suggests that not only the breed of chicken, but also the diet and even the breeding method, influence the composition of egg proteins. Previous experimental studies have shown that the addition of soybean meal and narrow-leafed lupin significantly affects amino acid composition. Therefore, manipulating feed composition could potentially also influence the immunoreactivity of these proteins [11].

The primary aim of this study was to evaluate whether changes in the protein source in hen diets may influence the immunoreactivity of selected egg allergens detected by ELISA. Additionally, the content of lysozyme was examined colorimetrically. The analysis was designed to determine whether manipulating the amount of soymeal, replacing some of the soymeal with lupine, or eliminating these legumes from the hens’ diet increased, decreased, or had no effect on egg allergenicity.

The second objective was to assess the limitations of ELISA for egg allergen detection. Enzyme-linked immunosorbent assay (ELISA) is recommended and the most commonly used method for the detection and quantitative analysis of food allergens due to its high sensitivity, ease of analysis, and the possibility of standardizing procedures [12]. However, a disadvantage of ELISA is that researchers often detect cross-reactivity with other matrix components, leading to false-positive results [12].

## 2. Materials and Methods

### 2.1. Nutritional Experiment on Hens

A four-week feeding study was conducted on 360 Hy-Line Brown hens aged 22 weeks. The birds were randomly assigned to cages and fed one of six experimental diets, each containing different proportions of soybean meal and ground low-alkaloid blue lupin (Sonet variety). A detailed description of the feeds (D1–DC) is provided in the Supplementary Materials in Table S2. The addition of narrow-leafed lupin and soymeal changed as follows: D1—15.43% soybean meal, 0% lupine; D2—11% soymeal, 10% lupine; D3—8.6% soymeal, 15% lupine; D4—5% soymeal, 20% lupine; D5—0% soymeal, 25% lupine; and DC—0% soy, 0% lupine (control diet). The study involved laying hens used for food production. The experimental design consisted exclusively of nutritional interventions, in which birds were fed diets differing in composition. No invasive or harmful procedures were applied, and animal welfare was not adversely affected. As part of the feeding experiment, hens were fed one of six diets with varying amounts of soybean meal and ground sweet narrow-leafed lupin seeds, prepared in accordance with the Poultry Nutrition Standards. The experiment was conducted in accordance with the Act of 15 January 2015 on the Protection of Animals Used for Scientific or Educational Purposes. The experiment did not require the approval of the Local Ethical Committee for Animal Experiments (Poland). Animals were housed and managed under standard husbandry conditions. The sex of the animals (female) was recorded and considered in the interpretation of the results where applicable. The experimental procedure was described in detail in a previous publication [11].

### 2.2. Antibodies

Six commercially purchased antibodies were used in the experiment (Table 1). In the ELISA analysis, 4 commercial antibodies directed against 3 egg white allergens (Gal d 1, Gal d 2, Gal d 3) and 1 antibody against Gal d 5—an egg yolk allergen—were used. Moreover, based on previously observed immunoreactivity, commercial anti-lupine and anti-soy antibodies were used. The antibodies (Table 1) were diluted according to the manufacturer’s information in 1% BSA in TBS pH 7.4. Mouse monoclonal anti-rabbit IgG, diluted 1:20,000, was used as the secondary antibody (A1949, Sigma Aldrich/Merck, St. Louis, MO, USA).

### 2.3. Standards

For egg proteins, three commercially available standards representing the major egg allergens were applied: chicken egg white ovomucoid (Gal d 1; trypsin inhibitor, T9253, Sigma-Aldrich, Merck, St. Louis, MO, USA), chicken egg white ovotransferrin (Gal d 3; conalbumin, C0755, Sigma-Aldrich, Merck), and chicken ovalbumin (Gal d 2; S7951, Sigma-Aldrich, Merck). For legumes, extracts were prepared from lupin (Sonet var.) and soybean meal (Augusta var.) at a 1:10 ratio in PBS buffer (pH 7.4), incubated overnight at 4 °C. The alpha-livetin standard, i.e., Gal d 5, is not commercially available; therefore, the standard used to create the curve was the control yolk.

### 2.4. Sample Preparation

Appropriate protein extracts were prepared from egg white and yolk, soybean, and lupin seed. Egg yolks and whites were separated and pooled to form a sample for analysis (~20 samples/group). The extraction of egg white and yolk was performed according to the methodology described by Tomczak et al. [10]. Egg white and yolk were extracted using phosphate-buffered saline (PBS) containing 0.05% Tween 20 (1:10, *v*/*v*), while soybean and lupin seed extracts were prepared in PBS (1:10, *v*/*v*). PBS was chosen to preserve the protein structure and ensure compatibility with antigen–antibody interactions in ELISA, as strong detergents can affect epitope accessibility and hinder immunochemical detection [13]. Samples were shaken for 18 h at 4 °C, then centrifuged at 13,400× *g*, and the supernatant was frozen at −20 °C for further analysis.

### 2.5. Commercial ELISA Kits

Three commercial tests were used to examine differences in the immunoreactivity of the albumen and yolk obtained in the experiment: I—Egg Test Kit, AgraQuant Plus, Romer Labs (Tulln, Austria), EG3018-43360, II—Lupin Test Kit, AgraQuant, Romer Labs, (Tulln, Austria), LU1014-1801; III—Soy ELISA Kit, EuroClone (Milan, Italy), EAE010096. The ELISA test was performed according to the leaflet included within the kit.

### 2.6. ELISA with Specific Antibodies

In order to determine the content of individual protein fractions, an indirect ELISA was chosen. The first step was to activate the plate with 100 µL of carbonate buffer (30 min/37 °C with mixing). To generate a calibration curve, 100 µL of sample/standard at a protein concentration of 1 mg/mL was added to microtiter plates, and a dilution series was prepared to obtain the optimal concentration range for generating the standard curve. The plates were incubated for 1 h 30 min at 37 °C. To check the concentration of allergen in egg samples, 100 µL of extract was applied to the plate. In further steps, the plates were washed with TBST buffer (TBS with the addition of 1% Tween 20). Blocking was performed using three blocking systems. For this, 1% casein and 1% gelatin in TBST were used to test the blocking systems in an ELISA using a chicken serum albumin antibody produced in rabbit as the primary antibody (the antibody manufacturer indicates cross-reactivity with BSA). The blocking agent used in the remaining ELISA analyses was 100 μL of 1% bovine serum albumin (BSA). Blocking was performed at 37 °C for 45 min. The plates were then washed again with TBST and incubated with the detection antibody according to the dilutions given in Table 1. After incubation at 37 °C for 1.5 h, plates were washed with TBST, and secondary antibodies were applied. After another wash and dry cycle, 100 µL of Ab II was added to the plate. The final reaction was induced using o-phenylenediamine (OPD, SIGMAFAST™, P9187 Merck, St. Louis, MO, USA) substrate (20 min in the dark) and then stopped by 2 M H_2_SO_4_. The absorbance was measured at λ = 492 nm using a UV-Vis spectrophotometer (Biochrom Asys UVM 340 Microplate Reader, Cambridge, UK).

### 2.7. Lysozyme Content

To determine lysozyme concentration, a standard curve was prepared using serial dilutions of lysozyme from hen egg white (≥90% purity, Sigma-Aldrich, St. Louis, MO, USA). Calibration solutions were prepared in the same extraction buffer as the analyzed samples to minimize matrix effects. Absorbance values obtained for the standards were used to generate a linear regression model, which was subsequently applied to calculate lysozyme content in the tested samples. Lysozyme concentration was expressed as mg/mL of egg white.

### 2.8. SDS-PAGE Electrophoresis

Sodium dodecyl sulfate–polyacrylamide gel electrophoresis (SDS-PAGE) was performed to compare the protein profiles of egg samples from different experimental groups. Samples were prepared according as previously presented [10]. Gels were stained using the Coomassie Brilliant Blue procedure.

### 2.9. Statistical Analysis

Each sample was analyzed using three technical replicates, and the mean value was used for further evaluation. Statistical analyses, using Statistica 14.1 software (StatSoft, Kraków, Poland), included the calculation of means, standard deviations, and comparison between experimental groups (ANOVA). In cases of significant differences, a post hoc analysis (Tukey’s test) was performed to determine homogeneous groups.

## 3. Results and Discussion

In the presented experiment, an ELISA method was used to monitor quantitative differences in the main egg allergen fractions, using commercial kits for allergen detection and six commercially available antibodies. Table 2 summarizes literature data on the relative abundance and approximate concentrations of the main egg allergens.

Table 3 presents the results obtained using commercial ELISA kits for detecting egg proteins and potential unidentified antibody reactions with soy and lupin proteins. A commercially purchased ELISA kit constructed for the content analysis of eggs in the food detected the highest reactivity of egg white from hens fed DC (117.16 mg/mL of egg white) and then D2 (113.17 mg/mL of albumen). The lowest value was recorded in eggs from D4 (51.60 mg/mL) and D3 (55.87 mg/mL) (Table 3). An approximately 50% increase in antibody response was observed in eggs depending on the hen’s diet. Statistical analysis of variance confirmed the significance of the observed differences (*p* = 0.0178).

The test was not suitable for the quantitative detection of yolk protein. Yolk samples contained substances preventing the correct detection of egg yolk allergens. These are expected to be protein–lipid fractions, which affected the stage of protein extraction and interaction with antibodies. Despite repeated dilution of the yolk samples, the threshold of quantitative detection was exceeded. Values exceeding the calibration are described in Table 3 as not possible for quantification (NQ). Additionally, in this study, commercial anti-lupin and anti-soy ELISA tests were used to analyze egg samples. None of the analyzed samples exceeded the limit of detection (Table 3). It informs us that both egg samples do not cross-react specifically with anti-legume antibodies and were not contaminated with soy or lupine. This confirms the observations made before (in Western blot analysis), concerning non-specific reactions of anti-legume antibodies with egg allergens. Moreover, these interactions depend on the applied experimental system of protein detection. After application of the same antibodies, but different extraction and detection systems, interactions are seen using Western blot analysis and not observed in ELISA tests.

The analysis confirmed that the obtained eggs differed significantly in immunoreactivity. A further step was to determine how the concentration of specific allergens in the tested eggs changed.

Table 4 presents the results of the analysis of individual egg allergen content in relation to the diet used, using the six available antibodies and the information on the lysozyme content.

### 3.1. Gal d 1

The highest content of ovomucoid using indirect ELISA was determined for eggs from the control diet (46.10 mg/mL of albumen) (Table 4). Similarly, a commercial test for these eggs (control diet) gave the highest result—117.16 mg/mL of white (Table 3). The lowest content of ovomucoid was detected in eggs from D1 (14.75 mg/mL of egg white), which is three times lower compared to the control diet. In the commercial test, the lowest content of immunoreactive protein was detected in sample D4 (51.60 mg/mL, Table 3). An analysis of variance (ANOVA) confirmed significant differences in ovomucoid content between dietary groups (*p* = 0.0144).

Commercial tests usually use ovomucoid epitopes for detecting the presence of eggs in food [7]. However, in the case of the test used, the manufacturer did not provide any information on the protein fractions detected during the analysis. The results of egg analysis with the anti-ovomucoid antibody and the commercial egg allergy test follow a similar trend. The content of allergen detected by the commercial test is much higher, indicating that this test may detect more than one egg protein or some other ovomucoid epitopes, but simultaneously detects those that are particularly ovomucoid. This is understandable, as ovomucoid is the allergenic protein most often causing food allergies to egg. Furthermore, ovomucoid is a highly resistant protein to both digestion and denaturation.

Researchers suggest a link between dietary protein and amino acid availability from a hen’s diet and egg protein synthesis. The effect of feed on egg protein content is neither simple nor linear, but there are several mechanisms through which nutrition can modulate the amount and structure of egg proteins. Ovomucoid synthesis requires an adequate supply of sulfur-containing amino acids (methionine, cysteine)—these are essential for the formation of disulfide bridges that stabilize the structure of this glycoprotein [19]. A diet low in methionine/cysteine can reduce the synthesis of proteins, because methionine is a limiting amino acid in poultry diets and must be supplemented. The supplementation of D4 in methionine was the highest [13]. There were no differences in the content of Met in the studied egg white, although the concentration of protein in albumen varied and was the highest for D4 albumen [13]. Thus, Met delivery by the diet is not an explanation for the observed differences in determined ovomucoid concentration. Conversely, a high-protein diet can increase total egg protein production and, consequently, the level of ovomucoid. In the experiment, the diets were balanced in protein content but could differ in protein availability. However, the differences in the ovomucoid content noted in the experiments between the studied eggs are surprisingly high.

It is important to note that the anti-ovomucoid antibody used is a polyclonal antibody. To verify the results, Western blotting studies were performed on these egg extracts (Figure S1, Supplementary Materials). This study indicated that the antibody recognized not only Gal d 1 but also Gal d 2 and Gal d 4, i.e., ovomucoid, ovalbumin and lysozyme, which may explain the higher quantitative results of the Gal d 1 content in the samples than those reported in the literature (Table 1). Therefore, the obtained values should be interpreted as differences in immunoreactivity detected by the anti-ovomucoid antibody rather than precise quantitative differences in ovomucoid concentration.

Nevertheless, as the same analytical conditions were applied to all samples, the results still allow relative comparison between dietary groups. Li et al., in their study conducted in [20], addressed the issue of ovomucoid assays. Purchased ovomucoid or ovalbumin standards are often contaminated, significantly complicating ELISA analysis. The authors point to the crucial use of a monoclonal antibody and a purified standard [20]. Testing the ovomucoid level using the ELISA method, which involves a complex matrix, is extremely difficult, even when using commercial tests intended for food samples. Ready-to-use ELISA kits for egg detection are optimized mainly for plant-derived food matrices, while the analysis of meat samples is more challenging due to matrix interferences that may decrease extraction efficiency and antibody binding [7].

### 3.2. Gal d 2

Table 3 contains information on the amount of ovalbumin in the tested samples. These values are approximately half of those reported in the literature. It should be noted that the values obtained in the present study reflect the immunoreactivity detected by ELISA rather than the absolute concentration of the proteins. The use of polyclonal antibodies and the complex composition of egg white may influence antibody binding and signal intensity. The highest ovalbumin contents were recorded in egg whites of hens fed D4 and DC—29.30 and 27.54 mg/mL of egg white, respectively. The lowest concentration recorded for D5 was 22.11 mg/mL and for D3 was 23.21 mg/mL (Table 4). The effect of the diet on the content of ovalbumin in eggs seems not to be as strong as in the case of ovomucoid. However, its significance has been confirmed in the ANOVA test (*p* = 0.0334).

Differences in the obtained results compared to the literature data can be explained by the extractant used or the extraction parameters (time, temperature, and multiplicity). Based on research conducted by Tomczak et al. 2021 TT—1% Tween 20 + 0.4% Triton X-100 is a suitable extractant for hen egg allergens for their simultaneous detection, but it was confirmed in qualitative (Western blot) analysis, and has not been checked in quantitative analysis [10]. The application of detergents may influence the extracted protein structure, which may not be suitable for qualitative analysis. The use of such reagents, i.e., strong detergents, in the ELISA technique is not recommended. In this study, we wanted to use one common extractant, PBS pH 7.4, which is the most frequently recommended extractant for the ELISA method, as well as for egg white proteins. In some cases, the buffered salt itself (such as PBS) may be insufficient and some modifications (surfactant, reductant) are needed, especially in the case of denatured proteins [21].

Ovalbumin is not very resistant to heat and proteolysis and therefore is frequently subjected to changes during food production. In addition, as in the case of ovomucoid, Western blotting analysis was performed to check antibody reactivity. These results are consistent with the ELISA results. A very weak detection of Gal d 2 in extracts by this antibody was observed, but the strongest response was observed for samples fed D4 and DC (Figure S1, Supplementary Materials). The studies described by Ruan et al. [22] indicate a relationship between feed enrichment with methionine and increased ovalbumin levels in egg whites of duck eggs. This is due to the key role of methionine in protein metabolism and synthesis, which directly translates into the quality and composition of eggs. Researching ovalbumin levels and attempts to control their levels has not only allergological but also industrial significance. Improving protein quality and increasing ovalbumin content influences functional properties, such as foaming and gelling ability. Ovalbumin is considered a key functional protein used in food technology [22].

### 3.3. Gal d 3

In studies of ovotransferrin concentration, the obtained results oscillate with the literature data (approx. 10–15 mg/mL of egg white) [18]. The lowest OVT content was determined in egg white from laying hens fed with the feed mixture marked as D4, and it was 10.16 mg/mL of egg white (Table 4). The highest result for ovotransferrin was recorded for egg protein obtained after the application of the control diet—18.27 mg/mL (Table 3). A ~45% increase in antibody response was observed depending on the diet. The observed differences have been confirmed by one-way analysis of variance (ANOVA) (*p* = 0.0301).

Many scientific studies over the years have examined the influence of various factors on Gal d 3 levels, but they do not address the hen’s diet. The level and structure of ovotransferrin are genetically encoded. Different breeds and lines of chickens may have different proportions of egg proteins, including ovotransferrin. In the case of ovotransferrin, genetics determines the amount of ovotransferrin in the protein, its precise structure, and even its susceptibility to denaturation (thermal change in structure). Therefore, ovotransferrin allergenicity is primarily a species-specific and individual characteristic. Dietary influence is considered secondary [23,24]. There are no studies in the literature that directly measure ovotransferrin (Gal d 3) levels in eggs depending on the protein source in the feed. On the contrary, its expression levels seem to be very stable [23,24]. However, our previous studies have shown differences in egg protein fractions in terms of immunoreactivity and the effect of changing the proportion of soymeal and lupin in the feed on the total protein content and amino acid composition of eggs, which may affect the protein ratio [10,11]. Although ovotransferrin expression is largely genetically determined, dietary factors may still influence its detected immunoreactivity. Changes in diet composition may affect overall protein synthesis in egg white or modify post-translational processes such as glycosylation, which can alter epitope accessibility and antibody binding.

### 3.4. Gal d 5

Gal d 5 cross-reacts with bird aeroallergens, such as blood serum or feathers. Furthermore, cross-reactivity with a protein present in egg white (Gal d 1) has been observed. People who are allergic to feathers, dander, or bird meat often cross-react with Gal d 5 [25].

Previous scientific papers have described difficulties with quantifying Gal d 5 in egg samples. Egg yolk is rich in lipids, proteins, and enzymes, which can interfere during ELISA (matrix effect). Due to this fact, without proper sample preparation (purification, appropriate dilution, and the removal of lipids), the ELISA signal can be distorted. Methodological and sequential work (e.g., the isolation and characterization of α-livetin) confirms the high homology of Gal d 5 with avian and mammalian albumins, including BSA, suggesting the possibility of cross-reactivity. The conclusion is that in immunoassays (e.g., ELISA), the use of BSA as a blocker or diluent may be problematic for egg allergens. Chicken serum albumin is present in all chicken tissues, such as blood and muscle tissue. Gal d 5 has also been identified as a cause of cross-allergy in chicken meat allergy [26]. For this reason, we encountered problems with the determination of Gal d 5 in the egg samples tested. Despite using three different blocking buffers and varying sample dilutions, up to 100,000-fold, we did not obtain a result that fell within the curve range. Sources indicate that commercial ELISA tests are not designed to analyze egg yolk proteins due to lipids that interfere with ELISA analysis. Hence, the inability to correctly detect yolk proteins using the commercial test could be observed. Western blot studies performed with this antibody indicate a very strong antibody response and confirm cross-recognition by the antibody of other allergens, such as Gal d 6, Gal d 2, and Gal d 4 (Figure S1, Supplementary Materials). This may additionally explain such a strong signal in the ELISA. Gal d 5 is serum albumin; numerous studies have shown that blood albumin concentrations in hens depend on feed composition (e.g., protein, amino acid levels, and additives). Therefore, dietary changes can alter plasma albumin concentrations, which could theoretically affect the amount of albumin transported into the yolk. This provides a logical mechanism but requires the empirical confirmation of a specific relationship with Gal d 5 in eggs [27].

### 3.5. Gal d 4

Lysozyme (peptidoglycan N-acetylmuramoyl hydrolase, EC 3.2.1.17) is a protein naturally occurring in egg white (albumen), considered a food allergen in individuals allergic to eggs. In the classification of egg allergens, lysozyme is described as a minor allergen [28]. The lysozyme content in the tested samples (Table 4) is consistent with the literature data [16]. The average lysozyme content in the tested samples was 3.24 mg/mL of egg white. The highest lysozyme content was recorded in egg white after the use of D1 and D3 (3.34 and 3.36 mg/mL white, respectively). The lowest content was recorded for samples after the control diet, 3.06 mg/mL white, and for the sample after D2—3.20 mg/mL. One-way analysis of variance (ANOVA) statistical analysis tested for significance in lysozyme content between dietary groups (*p* = 0.0226). Adding soymeal or narrow-leafed lupin increased lysozyme levels by approximately 10%. Although the differences in lysozyme concentration were statistically significant, the biological relevance of this difference may be limited and may reflect subtle variations in egg protein synthesis associated with dietary composition.

Multicomponent/total egg protein ELISA kits typically detect multiple egg proteins simultaneously (ovalbumin, ovomucoid, and very rarely, lysozyme). Most commercial ELISA kits detect the “egg in the product” via ovalbumin and ovomucoid antigens. Lysozyme and yolk proteins are rarely detected, only in broader-spectrum or more sensitive assays [7].

### 3.6. Reactivity of Yolk and Albumen Samples with Anti-Lupin and Anti-Soy Antibodies

At the end, due to the previously observed reactivity of anti-lupin and anti-soy antibodies with egg proteins, an ELISA test with monoclonal anti-lupine antibodies was also conducted. Similarly to the results of commercial tests, there was no detection of protein (Table 4), which confirms the absence of similar epitopes or the pollution of the studied samples. The lack of detectable reactivity with anti-soy and anti-lupin antibodies in this ELISA setup may be due to differences in assay conditions, epitope accessibility, and method detection limits.

### 3.7. SDS-PAGE Electrophoresis Results

The electrophoretic profiles of egg proteins did not reveal clear differences in the band patterns between the experimental groups. The major protein fractions appeared similar across all samples, suggesting that the dietary treatments did not result in substantial changes in the overall protein composition detectable by this method (Figure S2, Supplementary Materials).

In contrast, ELISA revealed differences in the immunoreactivity of selected egg allergens. This discrepancy may result from the fact that ELISA detects specific antigen–antibody interactions, which can be influenced by epitope accessibility or structural changes, rather than reflecting absolute protein content.

Therefore, the ELISA results should be interpreted with caution as indicators of changes in immunoreactivity rather than definitive quantitative differences in allergen levels.

On the other hand, the results presented were obtained after a commercial ELISA test was applied. These results confirm the variability in immunoreactivity between the tested samples; however, they are so significant that they suggest a deficiency in the available ELISA tests used to detect the presence of chicken eggs in food. Similarly, the antibodies used, selected with great care, did not allow for the quantitative identification of the tested allergens.

## 4. Conclusions

Innovative research on the impact of chicken nutrition on egg immunoreactivity is very difficult to conduct. The applied commercial kit, as well as the individual antibodies directed against the studied allergens, did not yield unambiguous quantitative results. However, observed differences in samples’ immunoreactivity, without a doubt, should arouse scientific interest and warrant continued investigation. From a public health perspective, diet-related differences in egg immunoreactivity may be relevant to risk assessment among egg-allergic consumers, especially given the relatively high prevalence of egg allergy in children.

The results suggest that dietary factors may influence the immunoreactivity of eggs detected by commercial ELISA tests. The interpretation of these findings should consider the methodological limitations of the applied immunochemical approach, including the inability to quantify Gal d 5 (due to detection limitations) and Gal d 6 (due to a lack of antibodies). The presented results should be interpreted primarily in terms of relative immunoreactivity rather than absolute protein quantification. The highest immunoreactivity (Gal d 1 and Gal d 3 content) is observed in eggs obtained after feeding with the control diet. A comprehensive analysis of the obtained data indicates that nutrition remains a potential tool for modulating egg immunoreactivity, although the degree of this modulation depends on the type of protein and its biological role.

## Figures and Tables

**Table 1:** Presentation of the used commercial detecting antibodies and their suggested dilution.

Commercial Antibodies	
I	Anti-Soy Protein antibody produced in rabbit, (S2519) Sigma Aldrich/Merck (St. Louis, MO, USA), dilution 1:5000
II	Anti-Conglutin gamma, lupin-specific globulin produced in rabbit, (AS08 335), Agrisera (Vännäs, Sweden), dilution 1:1000
III	Ovotransferrin Antibody—HRP Conjugated, produced in rabbit (OACA00203) Aviva Systems Biology (San Diego, CA, USA), dilution 1:2000
IV	Ovomucoid Antibody produced in rabbit, (OACA00210) Aviva Systems Biology (San Diego, CA, USA), dilution 1:2000
V	Ovalbumin Polyclonal Antibody, HRP, produced in rabbit, (PA1-196-HRP) ThermoFisher Scientific (Waltham, MA, USA), dilution 1:2000
VI	Chicken Serum Albumin antibody, produced in rabbit, (0118-70R-15097), Gentaur (Kampenhout, Belgium), dilution 1:2000

**Table 2:** Summary of information on the allergen content of chicken eggs.

Allergen (Name)	Symbol (Gal d)	Percentage of Egg Protein [14,15,16]	Approximate Concentration in Eggs
Ovalbumin	Gal d 2	~54%	~50–65 mg/mL of white [17]
Ovomucoid	Gal d 1	~10–11%	~8–12 mg/mL of white [14]
Ovotransferrin	Gal d 3	~12–14%	~10–15 mg/mL of white [18]
α-livetin (yolk protein)	Gal d 5	~7%	~1.1 mg/mL of yolk [15]
Lysozyme C	Gal d 4	~2–4%	~2.2–4.4 mg/mL of white [16]

**Table 3:** Results of egg allergenicity studies with commercially available ELISA kits [mg/mL of white or yolk].

Diet	Allergenic Proteins Content [mg/mL]					
	Egg Test Kit, AgraQuant Plus, RomerLabs		EuroClone Soy ELISA Kit		Lupin Test Kit, AgraQuant, Romer Labs	
	White	Yolk	White	Yolk	White	Yolk
D1	58.91 ± 0.31 ^c^	NQ	<0.002	<0.002	<0.002	<0.002
D2	113.17 ± 0.24 ^d^	NQ	<0.002	<0.002	<0.002	<0.002
D3	55.88 ± 0.11 ^b^	NQ	<0.002	<0.002	<0.002	<0.002
D4	51.60 ± 0.27 ^a^	NQ	<0.002	<0.002	<0.002	<0.002
D5	57.39 ± 0.21 ^c^	NQ	<0.002	<0.002	<0.002	<0.002
DC	117.16 ± 0.33 ^e^	NQ	<0.002	<0.002	<0.002	<0.002

Different letters in superscripts indicate statistically significant differences (*p* < 0.05) among values in each column. NQ—quantification not possible; D—diet of laying hens, containing: D1—15.43% soybean meal, 0% lupine; D2—11% soybean meal, 10% lupine; D3—8.6% soybean meal, 15% lupine; D4—5% soybean meal, 20% lupine; D5—0% soybean meal, 25% lupine; DC—0% soybean meal, 0% lupine (control diet).

**Table 4:** Egg allergen content in tested egg samples [mg/mL of white or yolk].

Diet	Gal d 1 (Ovomucoid)	Gal d 2 (Ovalbumin)	Gal d 3 (Ovotransferrin)	Gal d 5 (α-Livetin)	Soy	Lupine γ-Conglutin	Gal d 4 (Lysozyme)
D1	14.75 ± 0.47 ^a^ *	23.32 ± 0.03 ^b^	14.36 ± 0.46 ^d^	NQ	<0.002	<0.002	3.34 ± 0.03 ^b^
D2	33.07 ± 0.94 ^c^	27.40 ± 0.06 ^c^	12.08 ± 0.32 ^b^	NQ	<0.002	<0.002	3.20 ± 0.07 ^ab^
D3	32.66 ± 0.67 ^c^	23.21 ± 0.05 ^b^	13.31 ± 0.25 ^c^	NQ	<0.002	<0.002	3.36 ± 0.20 ^b^
D4	17.35 ± 0.41 ^b^	29.30 ± 0.03 ^d^	10.16 ± 0.26 ^a^	NQ	<0.002	<0.002	3.23 ± 0.07 ^ab^
D5	17.03 ± 0.36 ^b^	22.11 ± 0.07 ^a^	12.27 ± 0.27 ^b^	NQ	<0.002	<0.002	3.26 ± 0.04 ^ab^
DC	46.10 ± 0.65 ^d^	27.54 ± 0.08 ^c^	18.27 ± 0.21 ^e^	NQ	<0.002	<0.002	3.06 ± 0.05 ^a^

* Different letters in superscripts indicate statistically significant differences (*p* < 0.05) among values in each column. NQ—quantification not possible; D—diet of laying hens, containing: D1—15.43% soybean meal, 0% lupine; D2—11% soybean meal, 10% lupine; D3—8.6% soybean meal, 15% lupine; D4—5% soybean meal, 20% lupine; D5—0% soybean meal, 25% lupine; DC—0% soybean meal, 0% lupine (control diet).

## Data Availability

The original contributions presented in this study are included in the article/Supplementary Materials. Further inquiries can be directed to the corresponding author.

## Supplementary Materials

The following supporting information can be downloaded at: https://www.mdpi.com/article/10.3390/foods15071231/s1, Table S1: List of chicken egg allergens; Table S2: Composition of feed prepared for a feeding experiment conducted for 6 groups of laying Hy-Line hens [%]; Figure S1: Images of membranes after Western blot analysis using commercial antibodies. The main allergenic fractions of egg white and egg yolk are marked on the membranes (orange arrow). MW—molecular weight, W1–5—white extracts, Y1–5—yolk extracts, C—control group. I—Chicken Serum Albumin antibody, II—Ovalbumin Polyclonal Antibody, III—Ovomucoid Antibody, IV—Ovotransferrin Antibody, V—Anti-Conglutin gamma, lupine-specific globulin, VI—Anti-Soy Protein antibody. Figure S2: Electropherogram after separation of egg protein lysates using SDS-PAGE electrophoresis. W—egg white, Y—egg yolk, 1–5—experimental diet mixtures used in hen feeding (with variable amounts of soy and narrow-leafed lupine), C—control; MW—molecular weight marker.

## Abbreviations

D1	diet of laying hens with the addition of legumes: 15.43% soy, 0% lupine
D2	diet of laying hens with the addition of legumes: 11% soy, 10% lupine
D3	diet of laying hens with the addition of legumes: 8.6% soy, 15% lupine
D4	diet of laying hens with the addition of legumes: 5% soy, 20% lupine
D5	diet of laying hens with the addition of legumes: 0% soy, 25% lupine
DC	diet of laying hens without the addition of legumes: 0% soy, 0% lupine
