# Supplementary material for: Feed Components and Egg Allergenicity: Impact of Lupin and Soybean Meal Inclusion on Hen Egg Immunoreactivity (ELISA-Based Study)
Source: Foods. 2026 Apr 4;15(7):1231. doi: 10.3390/foods15071231 (PMC13072771; doi:10.3390/foods15071231)
Supplement: Supplementary file 1 [file foods-15-01231-s001.zip › foods-4186532-supplementary.pdf]

Table S1. List of chicken egg allergens

Allergen name	Biochemical name	MW (kDa)	Allergenicity references (https://www.allergen.org/index.php)	Another information [1–3]	Content in egg [%] [4]
**Gal d 1**	Ovomucoid, a trypsin inhibitor (OVM/OM)	28	Nearly 100% of people with egg allergy are allergic to ovomucoid (32/33 patients).	Found only in egg whites, it is the most important egg allergen. Ovomucoid is a glycoprotein. It inhibits trypsin (a digestive enzyme), helping to protect the egg's contents from bacteria. It is very heat-stable; heat treatment (boiling, frying, baking) does not completely destroy it. It is also resistant to digestion, which is why it is often responsible for persistent egg allergies, especially in young children.	11 % of egg white
**Gal d 2**	Ovalbumin (OVA)	44	All patients with egg protein allergy studied were allergic to Gal d 2.	It is the most abundant protein in chicken eggs. It is a globulin belonging to the serpin family (protease inhibitors), although it does not act as an enzyme inhibitor like other serpins. In eggs, it serves primarily as a source of amino acids for the developing embryo. It is more temperature-sensitive than ovomucoid – it denatures easily during cooking, which affects the consistency of the egg white after processing. It is relatively easily digested. Ovalbumin is the egg allergen Gal d 2, but it is less stable than ovomucoid, and allergy to it tends to decrease with age. People allergic to OVA often tolerate cooked eggs. Gal d 2 exhibits strong	54% of egg white

				cross-reactions, primarily with milk α-lactalbumin and poultry meat. It is the second most common egg white allergen.	
**Gal d 3**	Ovotransferrin, an iron-binding protein (KB, conalbumine)	78	94% of the examined patients (31/33) with egg allergy showed IgE binding to Gal d 3.	Ovotransferrin is a glycoprotein that strongly binds Fe^{3+}, as well as other metals such as Cu^{2+} and Zn^{2+}, thus serving as an antibacterial agent. It exhibits bactericidal properties, primarily against Gram-negative bacteria, and to a lesser extent against Gram-positive bacteria. It is the third allergen in egg white that most commonly causes allergic reactions, but ovotransferrin is relatively temperature-sensitive.	12% of egg white
**Gal d 4**	Lysozyme C	14	Of the more than 30 egg allergy sufferers tested, as many as 22 (67%) had reactions to lysozyme.	Lysozyme is a protein enzyme that hydrolyzes chemical bonds, and according to its nomenclature, it is N-acetylmuramidoglycanohydrolase. This enzyme is responsible for breaking down the glycosidic bond between N-acetyl-D-glucosamine and N-acetylmuramic acid in murein, a component of the capsule of Gram-positive bacteria. Lysozyme protects the embryo from bacterial infection. It is relatively resistant to temperature and pH, but prolonged cooking leads to denaturation. Allergy to Gal d 4 is rare.	3-4% of egg white
**Gal d 5**	α-livetin/serum albumin	69 kDa	All patients with food allergy to egg yolk	Alpha-livetin is an egg yolk serum protein. It belongs to	40-60% of egg yolk

			studied had reactions to Gal d 5.	the globulin group and is present in smaller quantities than the main egg white proteins. Alpha-livetin participates in the transport of nutrients (amino acids, lipids) to the developing embryo. It is found not only in egg yolk but also in feathers and serum. This allergenic fraction is responsible for cross-reactions with meat and feathers. Designated as Gal d 5, it is responsible for allergic reactions, primarily to raw egg yolk. Heat treatment may partially reduce its allergenicity.	
**Gal d 6**	YGP42, i.e., the precursor of vitellogenin 1, β-livetin, yolk glycoprotein 42	35 kDa	The apparent mass of YGP42 is 35 kDa due to the naturally occurring glycosylation of this protein. 18% of patients with egg yolk allergy exhibit Gal d 6 allergy in addition to Gal d 5 allergy.	YGP42 is an egg yolk glycoprotein (42 kDa). It belongs to the lipoprotein/yolk serum glycoprotein group. It is designated in the allergen classification as Gal d 6. It participates in the transport of nutrients, particularly lipids, to the developing embryo. Heat treatment may reduce its allergenicity, but reactions may still occur in allergic individuals.	nd

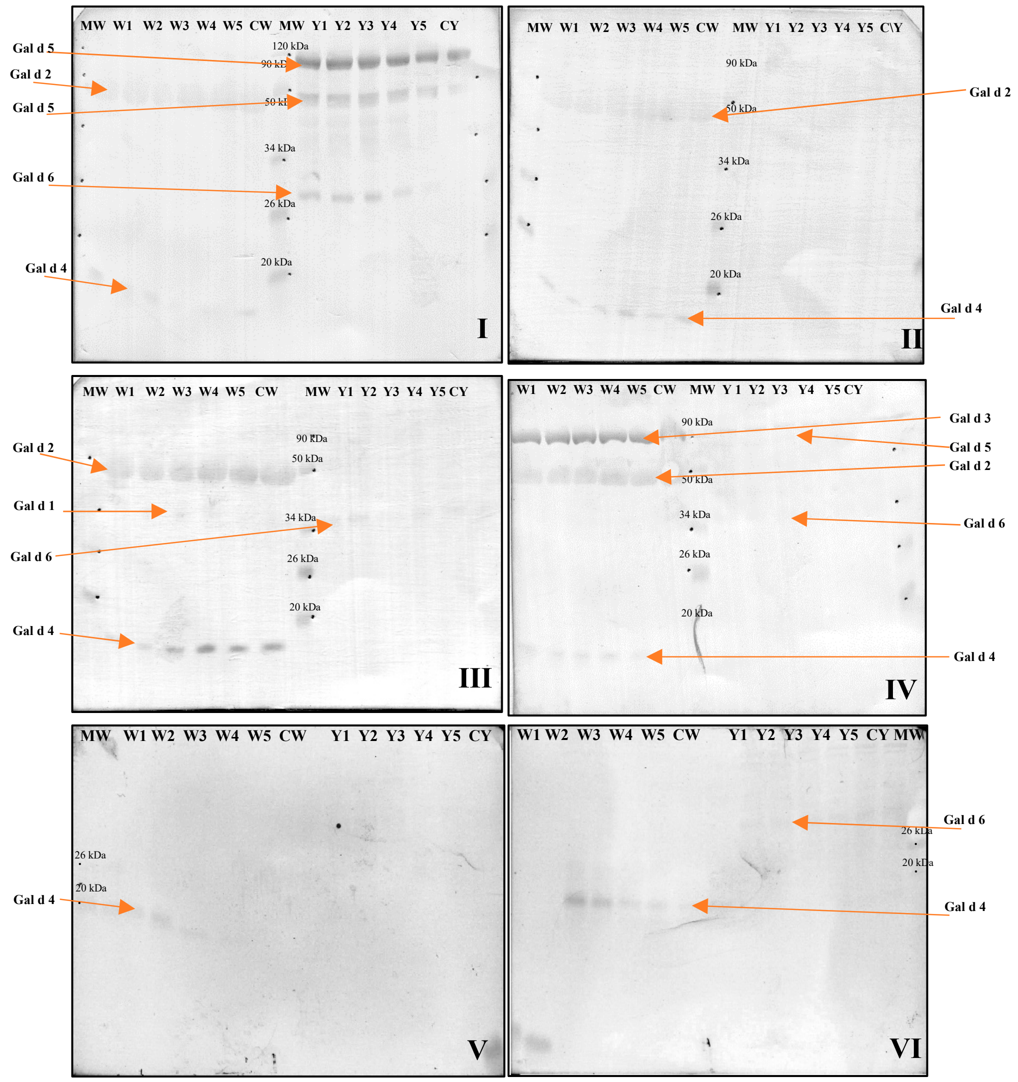

Figure S1. Images of membranes after Western blot analysis using commercial antibodies. The main allergenic fractions of egg white and egg yolk are marked on the membranes (orange arrow). MW – molecular weight, W1-5 – white extracts, Y1-5 – yolk extracts, C – control group. I - Chicken Serum Albumin antibody, II - Ovalbumin Polyclonal Antibody, III - Ovomucoid Antibody, IV - Ovotransferrin Antibody, V - Anti-Conglutin gamma, lupine-specific globulin, VI - Anti-Soy Protein antibody

Table S2. Composition of feed prepared for a feeding experiment conducted for 6 groups of laying Hy-Line hens [%].

	Treatment					
**Component %**	**D1**	**D2**	**D3**	**D4**	**D5**	**DC**
**Wheat (CP 118)**	58.15	53.67	50.28	48.37	47.98	30.00
**Corn (CP 94)**	-	-	-	-	-	23.19
**Soybean meal**	15.43	11.00	8.60	5.00	-	-
**Blue lupine**	-	10.00	15.00	20.00	25.00	-
**Peas**	10.00	10.00	10.00	10.00	10.00	10
**Rapeseed oil**	4.15	4.70	5.50	6.00	6.30	6.02
**Limestone (fine to coarse - 40:60)**	9.15	8.10	8.10	8.05	8.06	9.84

**Rapeseed meal (CP 349)**	-	-	-	-	-	5.00
**Sunflower meal (CP 340)**	-	-	-	-	-	5.00
**Corn gluten**	-	-	-	-	-	5.00
**Potato protein**	-	-	-	-	-	2.00
**Mono calcium phosphate**	1.28	1.28	1.29	1.30	1.31	1.52
**Premix 0.5%**	0.50	0.50	0.50	0.50	0.50	1.00
**NaCl**	0.18	0.19	0.20	0.20	0.19	0.61
**NaHCO3**	0.35	0.30	0.29	0.29	0.29	0.27
**DL-Methionine (98%)**	0.15	0.15	0.15	0.20	0.15	0.15
**HCl-lysine (78%)**	0.15	0.02	-	0.01	0.08	0.35
**L-Threonine (98%)**	0.05	0.02	0.02	0.01	0.04	0.02
**L-Tryptophan (98%)**	-	-	-	-	-	0.03
**L-Valine (98%)**	0.10	0.07	0.07	0.07	0.10	-
**Metabolizable Energy [MJ/kg]**	11.61	11.62	11.68	11.64	11.62	11.80
**Components %**						
**Crude protein**	16.36	16.42	16.43	16.49	16.41	17.02
**Ca**	3.5	3.53	3.52	3.5	3.5	4.3
**P-available**	0.39	0.39	0.39	0.39	0.39	0.46
**Na**	0.18	0.17	0.17	0.18	0.18	0.24
**Cl**	0.18	0.16	0.16	0.17	0.17	0.46
**Lys.** ***digest.***	0.76	0.75	0.75	0.75	0.75	0.69
**Met+Cys*digest.***	0.65	0.64	0.65	0.67	0.65	0.62
**Thr*digest.***	0.53	0.53	0.54	0.54	0.54	0.49
**Try*digest.***	0.16	0.17	0.17	0.17	0.17	0.15
**Val** ***Tot.***	0.71	0.71	0.72	0.72	0.72	0.98
**Arg*Tot.***	0.98	1.25	1.34	1.43	1.52	0.92
**Linoleic acid**	1.73	1.67	1.84	2.01	2.16	1.79

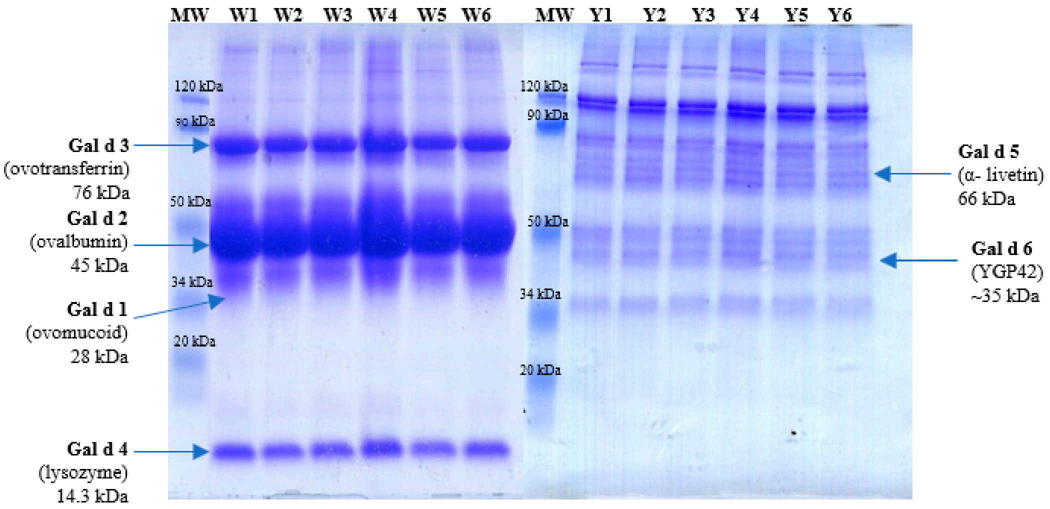

Figure S2. Electrophoregram after separation of egg protein lysates by SDS-PAGE electrophoresis. W – egg white, Y – egg yolk, 1-5 - experimental diet mixtures used in hen feeding (with variable amounts of soy and narrow-leafed lupine), C – control; MW—molecular weight marker.
